# Usefulness of partial splenic embolization for left-sided portal hypertension in a patient with a pancreatic neuroendocrine neoplasm: a case report and review of the literature

**DOI:** 10.1007/s12328-022-01631-7

**Published:** 2022-04-16

**Authors:** Teppei Matsui, Hidenari Nagai, Makoto Amanuma, Kojiro Kobayashi, Yu Ogino, Takanori Mukozu, Noritaka Wakui, Naoki Okano, Yoshinori Kikuchi, Takahisa Matsuda, Yoshinori Igarashi

**Affiliations:** Division of Gastroenterology and Hepatology, Department of Internal Medicine (Omori), School of Medicine, Faculty of Medicine, Toho University, 6-11-1, Omorinishi, Ota-ku, Tokyo, 143-8541 Japan

**Keywords:** Partial splenic embolization, Left-sided portal hypertension, Pancreatic neuroendocrine neoplasm

## Abstract

Left-side portal hypertension (LSPH) is caused by isolated obstruction of the splenic vein and is associated with esophagogastric varices that extend from the lower esophagus to the greater curvature of the gastric body. Here, we report on a 74-year-old man with a pancreatic neuroendocrine neoplasm (NEN) in the pancreatic tail with multiple liver metastases. We decided that partial splenic embolization (PSE) was the best course of treatment to prevent rupture of the gastric varices, which were classified as markedly enlarged, nodular, or tumor-shaped and showed erosion of the mucosa. After PSE, the patient had no major complications and was discharged. At 3 and 6 months after the procedure, esophagogastroduodenoscopy and enhanced computerized tomography showed that the gastric varices had improved. This case demonstrates the usefulness of PSE for LSPH in patients with unresected pancreatic NEN.

## Introduction

Transcatheter partial splenic embolization (PSE) is an effective alternative to splenectomy and is less invasive [1–4]. PSE not only increases the platelet count but also ameliorates portal hypertension and reduces esophageal varices by decreasing blood flow from the splenic vein [5]. The reduced blood flow from the enlarged spleen subsequently decompresses gastric and duodenal bulb varices. Previous studies found that PSE is an effective method for controlling bleeding from gastric and duodenal varices secondary to splenic vein obstruction [6, 7].

Left-side portal hypertension (LSPH), also known as sinistral hypertension, is a rare but life-threatening cause of upper gastrointestinal bleeding. It is caused by the isolated obstruction of the splenic vein and is associated with esophagogastric varices extending from the lower esophagus to the greater curvature of the gastric body [8]. Here, we present a patient with LSPH due to an unresected pancreatic neuroendocrine neoplasm (NEN). The case demonstrates the usefulness of PSE.

## Case report

In 1999, at the age of 57 years, the patient was diagnosed with a neuroendocrine neoplasm (NEN) of the pancreatic tail with multiple liver metastases at another hospital. At the initial diagnosis, the patient did not wish to undergo surgical treatment or systemic chemotherapy. Therefore, selective hepatic transcatheter arterial embolization (TAE) was performed for the metastatic liver lesions as palliative care, which may be a possible prognostic factor in this case. In 2013, he was diagnosed with bone and spleen metastasis, and the primary tumor was found to be enlarged. In 2016, he had epigastric pain and was examined by esophagogastroduodenoscopy, which identified gastric varices. Therefore, he was referred to our hospital for treatment of the varices.

**Fig. 1:**
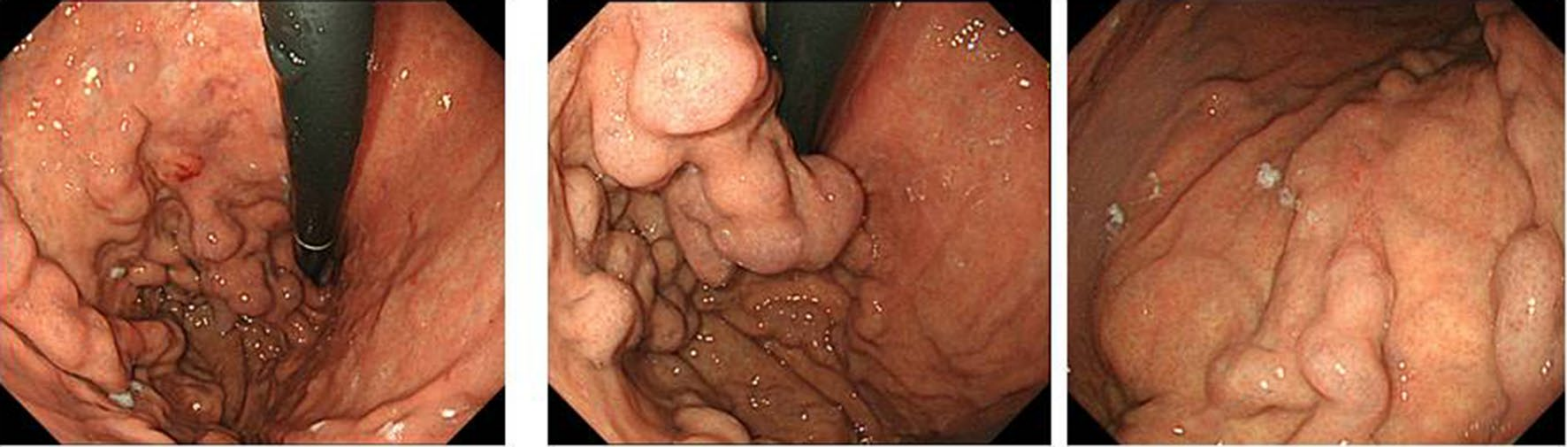
Esophagogastroduodenoscopy on admission. Gastric varices were seen in the fundus of the stomach extending from the cardiac orifice to the fornix and in the gastric body

**Fig. 2:**
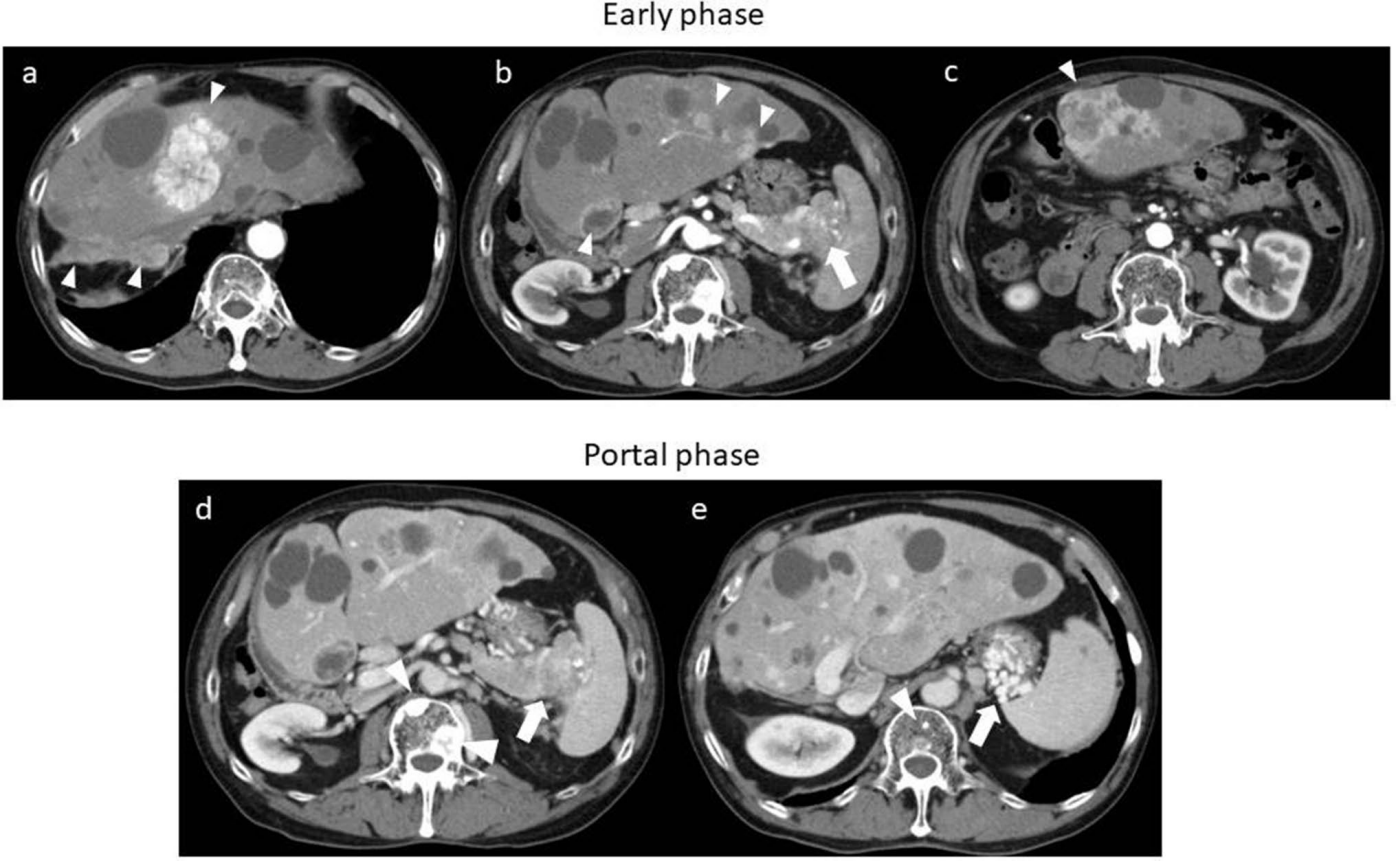
Enhanced computerized tomography on admission. In early phase, multifocal liver metastases were found in both lobes (**a**, **b**: arrowhead). In particular, the lateral area of the left lobe showed diffuse infiltration (**c**: arrowhead). The pancreatic NEN was seen into the splenic hilum (**b**: arrow). In portal phase, enlargement of the short gastric vein and disruption of the splenic vein (**d**, **e**: arrow). The multiple metastatic spinal tumors were also observed (**d**, **e**: arrowhead)

On admission in July 2016, at the age of 74 years, his height was 167.5 cm; weight, 65 kg; temperature, 35.8 °C; blood pressure, 136/64 mmHg; and pulse rate, 84/min. There were no symptoms related to excessive hypersecretion of hormones and/or monoamines. Examination of the abdomen revealed no tenderness, abnormal masses, or organomegalies. Laboratory tests revealed a low hemoglobin concentration (13.0 g/dl) and platelet count (10.2 × 10^4^/μl) and showed mild elevation of aspartate aminotransferase (58 IU/l) and gamma-glutamyl transpeptidase (177 IU/l). Esophagogastroduodenoscopy revealed gastric varices (GV) in the fundus of the stomach (extending from the cardiac orifice to the fornix and in the gastric body; markedly enlarged, nodular, or tumor-shaped; white; no red color sign; and showing erosion of the mucosa) (Fig. 1). An abdominal–pelvic enhanced computerized tomography (CT) scan with intravenous contrast revealed enlargement of the short gastric vein and disruption of the splenic vein due to invasion of the pancreatic NEN into the splenic hilum, as well as slight splenomegaly (Fig. 2b, d, e: arrow). In addition, multifocal liver metastases were found in both lobes. In particular, the lateral area of the left lobe showed diffuse infiltration (Fig. 2a, b, c: arrow head), and multiple metastatic spinal tumors were also observed (Fig. 2d, e: arrow head).

In July 2016, we performed a liver tumor biopsy to determine the course of treatment and diagnosed a neuroendocrine tumor (NET), G1. The patient was diagnosed with gastric varices associated with LSPH due to the pancreatic non-functional NEN.

**Fig. 3:**
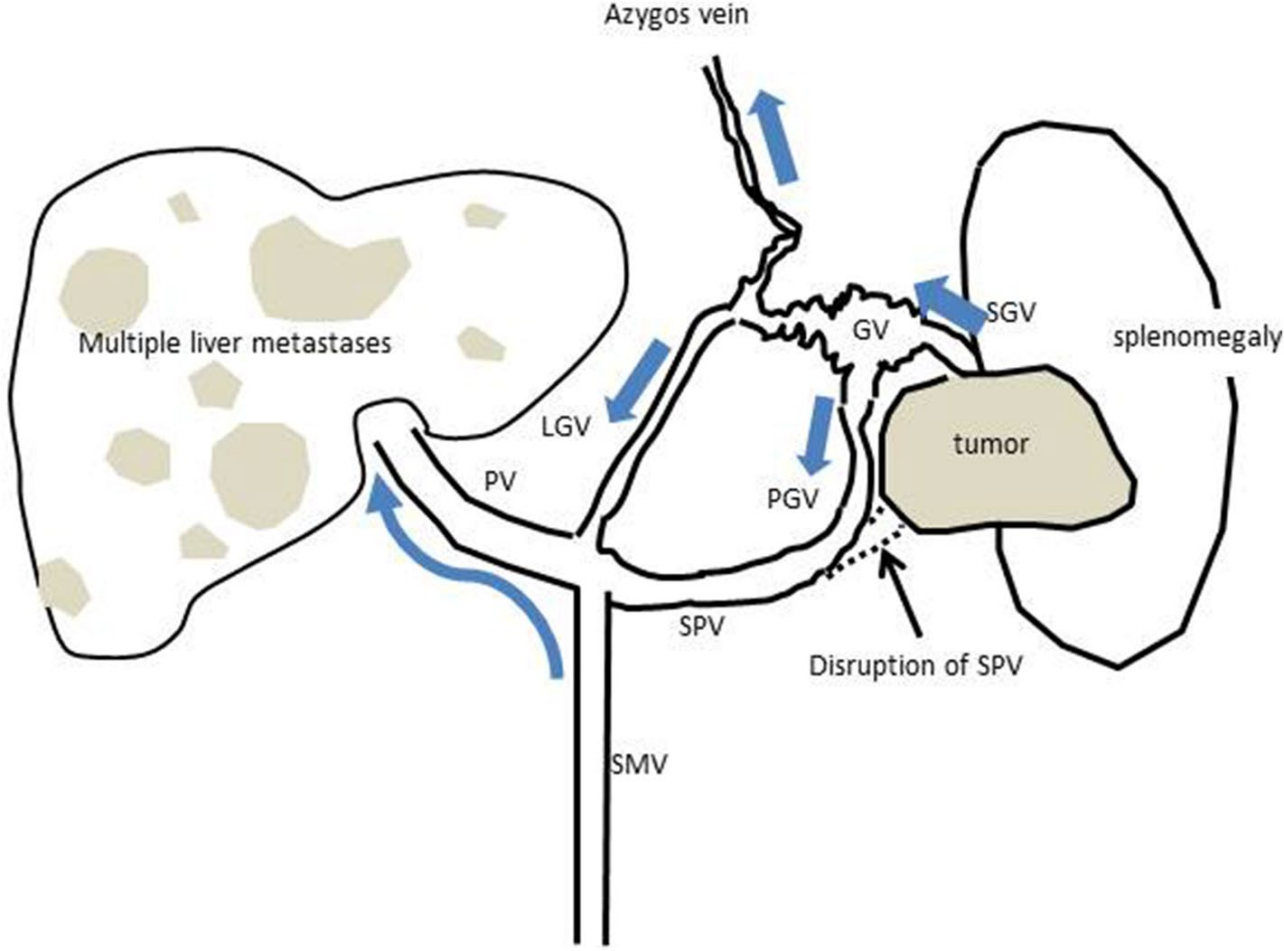
The hemodynamic schema of LSPH in this case. *LGV* left gastric vein, *PGV* post-gastric vein, *SGV* short gastric vein, *PV* portal vein, *SMV* superior mesenteric vein, *GV* gastric varices

Although his performance status was maintained (ECOG PS; 0), the stage of disease was advanced and radical resection was considered impossible. Furthermore, removal of the spleen may also not have been successful because of severe infiltration into the splenic hilum. As a result of the hemodynamic study, the GV was found to be supplied by the short gastric vein (SGV), and the draining veins were (1) the azygos vein flowing into superior vena cava, (2) the post-gastric vein (PGV), and (3) the left gastric vein (LGV) flowing into portal vein (Fig. 3). Blocking the SGV using percutaneous transhepatic obliteration (PTO) was thought to be impossible because of the obstruction of the splenic vein. In addition, blocking the drainage route using PTO or balloon occluded retrograde transvenous obliteration (BRTO) was unsuitable because of the high possibility of inadequate treatment due to the existence of three drainage routes (LGV, PGV, azygos vein). Endoscopic treatment was also considered unsuitable as the GV were widespread in the gastric body. Therefore, we decided that PSE was the best course of treatment to prevent rupture of the gastric varices, and we performed PSE in July 2016.

**Fig. 4:**
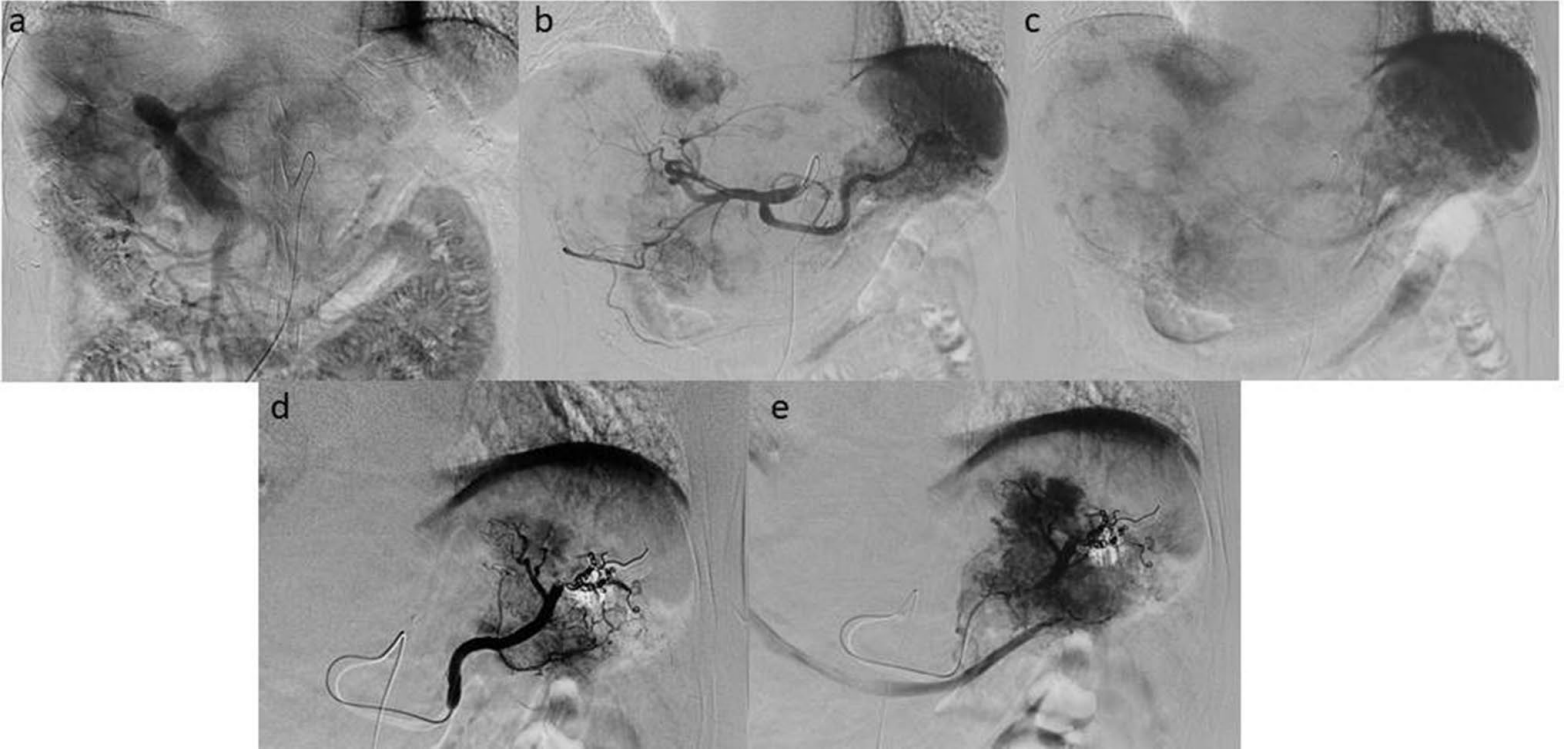
Arteriography and transcatheter partial splenic embolization. On transarterial portogram from SMA, main tract of portal vein was observed without any growth of collateral veins (**a**). On celiac angiogram, multiple tumor stain in both hepatic lobes, and splenomegaly was seen (**b**). On transarterial portogram from SPA, collateral vein was developed as SGV (**c**) forming GV. Microcoils were used to embolize the branches of the SPA to achieve embolization rate of 60% (**d**, **e**). *SMA* superior mesenteric artery, *SPA* splenic artery, *GV* gastric varices, *SGV* short gastric vein

**Fig. 5:**
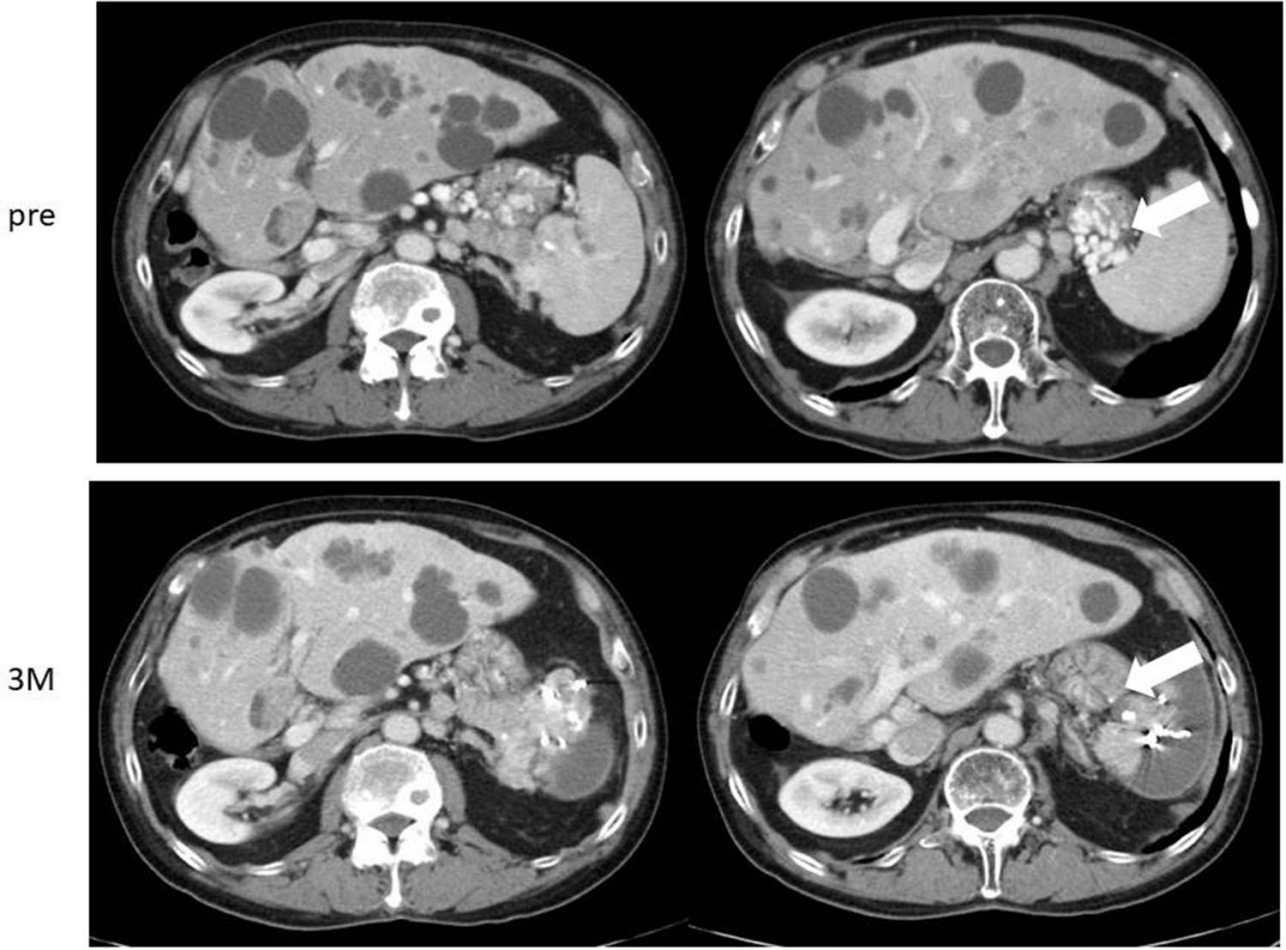
The comparison of enhanced computerized tomography before and after PSE. The disappearance of enlargement of the SGV and GV are shown (arrow)

**Fig. 6:**
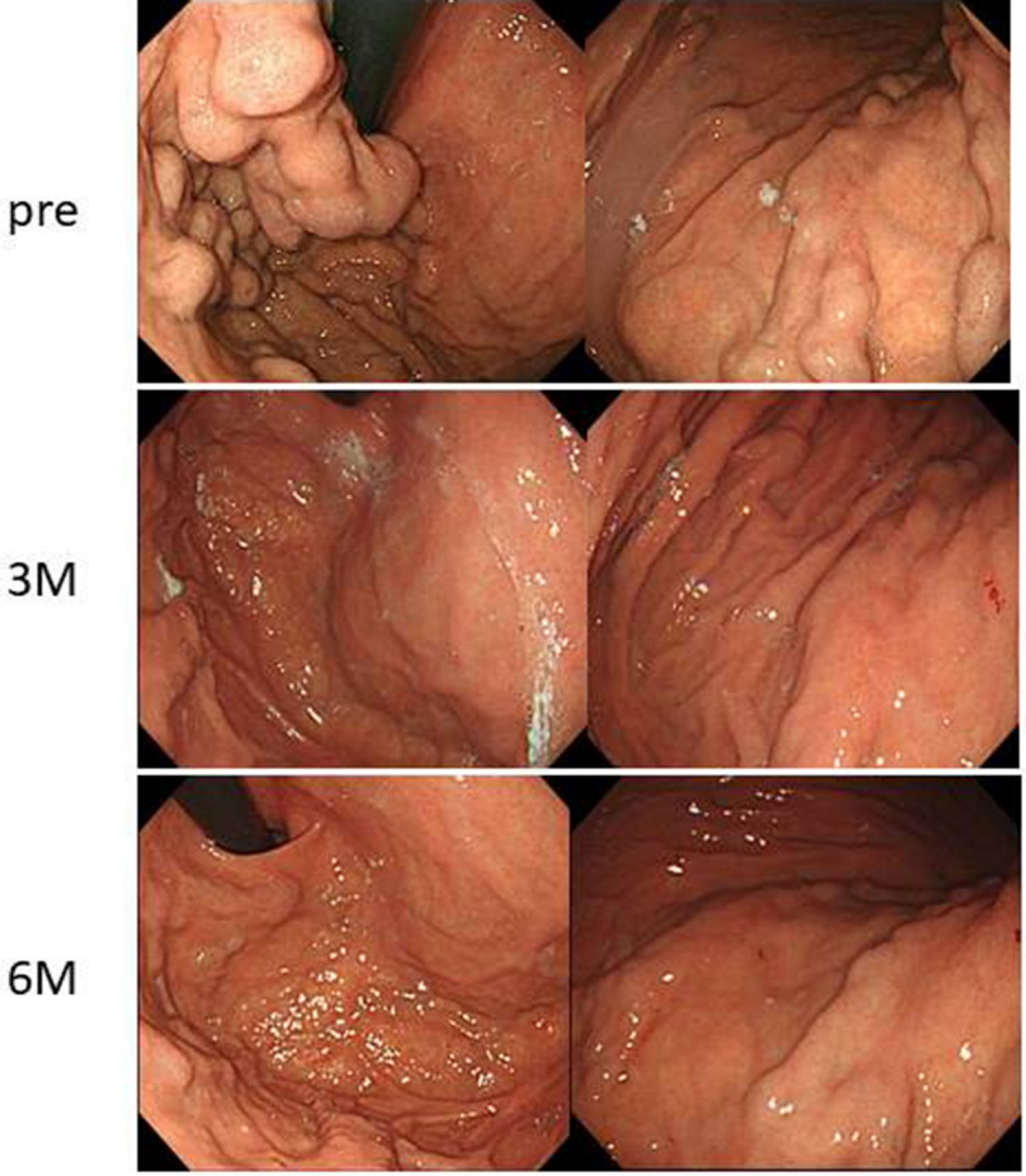
Esophagogastroduodenoscopy after PSE. Improvement of the GV is shown

For PSE, a catheter was inserted into the right femoral artery under local anesthesia with 1% lidocaine and was advanced to the hilum of the splenic artery. Before PSE, arteriography was performed, and the pre-embolization splenic arteriogram showed that the splenic vein had completely disappeared, the short gastric vein was enlarged, and the tumor was slightly dense in the hilum of the spleen (Fig. 4b, c). Then, branches of the splenic artery were embolized with microcoils and pieces of gelatin sponge, with the aim to achieve about 60% embolization of the spleen [9]. After PSE, a splenic arteriogram showed decreased blood flow in the short gastric vein (Fig. 4d, e). The patient had no major complications and was discharged. Improvement of the gastric varices was shown by enhanced CT at 3 months after PSE (Fig. 5: arrow), and by esophagogastroduodenoscopy at 3 and 6 months after PSE (Fig. 6).

**Fig. 7:**
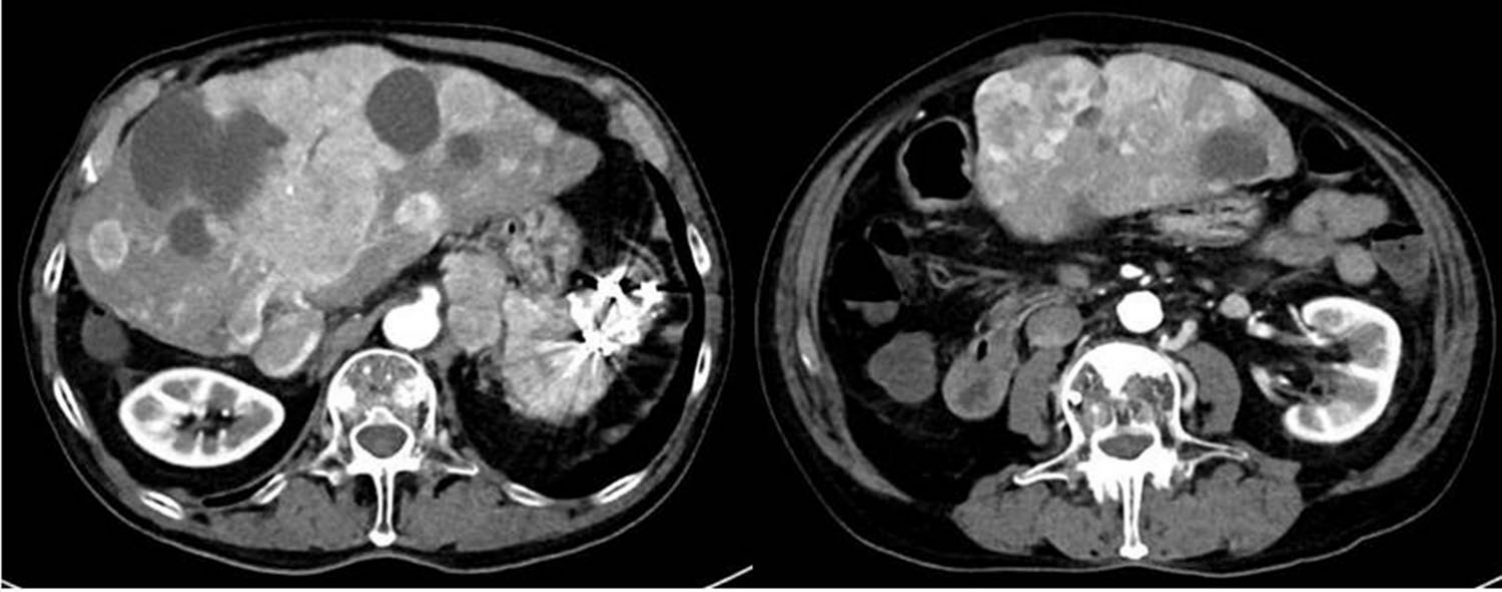
Enhanced computerized tomography at March 2018. The NEN was enlarged, and both lymph nodes and liver metastatic lesions were advanced

After PSE, we recommended medical therapy with somatostatin analogs (SSA) as antiproliferative treatment, but he did not agree to treatment for financial reasons. Finally, in March 2018, he agreed to undergo medical therapy after long deliberation, and we started octreotide LAR administration. However, the hepatic metastatic lesions grew to occupy a large part of the liver, and both the pancreatic NEN and metastases of lymph nodes were enlarged (Fig. 7). The patient’s general condition gradually deteriorated, but no gastrointestinal bleeding occurred, and he died of liver failure and cachexia in December 2019.

## Discussion

In European and US referral centers, up to 77% of patients with pancreatic NEN and up to 91% of patients with intestinal NEN present with distant metastases at the initial diagnosis [10–13]. Most NEN are non-functional and discovered incidentally or due to unspecific symptoms [14]. In the present case, the patient had multiple liver metastases at the time of initial diagnosis, and he had no symptoms related to excessive hypersecretion of hormones and/or monoamines. In the most recent Surveillance, Epidemiology, and End Results (SEER) database analysis, the median survival for distant metastatic disease was 33 months in patients with NET G1-G2, and survival at five years was 35% in well-differentiated to moderately differentiated NET [15]. According to the national database and NET registries, it appears that prognosis has improved with the 5-year overall survival increasing up to 60% in patients with metastatic pancreatic NET undergoing multidisciplinary treatment [14]. Although the prognosis of pancreatic NEN is thought to be prolonged by the development of therapies, this case is rare because the patient survived for a very long time (20 years) after the initial diagnosis. In addition to the above, there are no similar reports of successful PSE for gastric varices associated with LSPH due to pancreatic NEN; hence, we present this case.

Left-side portal hypertension (LSPH), or sinistral portal hypertension (SPH) is a rare cause of upper gastrointestinal hemorrhage. The main characteristic of LSPH is gastric variceal hemorrhage due to splenic vein thrombosis or occlusion. LSPH is caused by pancreatic diseases, such as acute or chronic pancreatitis and pancreatic pseudocysts and carcinomas [16–19]. It is a rare form of portal hypertension that accounts for approximately 5% of cases; it occurs within the left gastrosplenic region and is primarily caused by thrombotic occlusion of the splenic vein [20]. Madsen MS et al. reported that in 65% of cases the cause of LSPH was pancreatitis and that 33% of patients with LSPH had a pancreatic pseudocyst. Benign and malignant pancreatic tumors were the cause of splenic vein obstruction in 18% of patients, and a number of different diseases were the cause in 17% of patients. Furthermore, the group reported that the most common symptom (in 75% of patients) was bleeding from gastroesophageal varices and that in 75% of patients the conditions in the esophagus and stomach were evaluated by angiography, upper endoscopy, and/or perioperative findings [8]. Our patient was diagnosed with LSPH due to disappearance of the splenic vein because of invasion of a pancreatic NEN located in the pancreatic tail. The patient required treatment to prevent rupture of the markedly enlarged, nodular, or tumor-shaped gastric varices with mucosal erosion. We chose PSE as a treatment for gastric varices based on the hemodynamic diagnosis and to maintain the patient’s quality of life (QoL) for as long as possible. PSE was performed without any complications, and we succeeded in preventing bleeding from the gastric varices, improving the patient’s QoL.

Surgical resection with curative intent remains the gold standard for treating liver metastasis, achieving a 60–80% survival rate at 5 years with low mortality (0–5%) and acceptable morbidity (close to 30%) [14]. In addition, the guideline by the Japan Neuroendocrine Tumor Society (JNETS) recommends that pancreatic and gastrointestinal NET with liver metastasis are considered eligible for hepatic resection when there is no extrahepatic metastasis [21]. The present case was complicated by multiple bone metastases in 2016, which is not an indication for curative resection and is an indication for multidisciplinary treatment according to the JNETS algorithm.

We introduced medical therapy with somatostatin analogs (SSA) as an antiproliferative treatment in March 2018. SSA, especially octreotide LAR, is recommended for anti-proliferative purposes in both functioning and non-functioning mid-gut tumors [14]. The patient continued to receive octreotide LAR for 1.5 years without any symptoms. Although both the pancreatic NEN and metastatic lesions grew, the patient’s QoL was maintained until the end-stage of his life. In addition, the patient survived for a very long period of time, 20 years from the initial diagnosis, and it is thought that the prevention of life-threatening gastrointestinal bleeding from GV by PSE contributed to the long-term survival of this patient.

We performed a literature search in PubMed with the keywords “neuroendocrine” and “sinistral portal hypertension” for the years 2001–2021 and identified three publications of case report [22–24]. However, when we included the keyword “partial splenic embolization” we did not identify any papers. Therefore, we assume that PSE is a rare treatment for LSPH caused by a pancreatic NEN in the pancreatic tail.

Interestingly, although most studies on LSPH and pancreatic tumors are case reports or small series, Moyana et al. reported a retrospective review of the association between pancreatic NEN and sinistral portal hypertension in 61 patients with pancreatic NEN [25]. This retrospective review reported that 8 of the 61 pancreatic NEN cases had a splenic vein thrombosis and GV at the time of diagnosis. Furthermore, they reported that four of these eight cases met the strict criteria for sinistral portal hypertension, and three of these four cases presented with bleeding from GV. They emphasize the usefulness of surgical resection; even in patients where curative resection is not possible. Moreover, palliative resection with splenectomy can improve the quality of life and outcomes. Based on Moyana’s report, GV caused by pancreatic NEN with sinistral portal hypertension are considered to have a high risk of bleeding. Therefore, it is necessary to consider surgical treatment, including palliative resection with splenectomy, even in those cases where radical resection is not possible, to prevent bleeding from GV. However, as surgical treatment is highly invasive, the less invasive PSE may be useful, after careful consideration of hemodynamics, as initial treatment in cases where radical resection is not possible.

## Conclusion

Our experience of treating gastric varices by PSE in a patient with LSPH due to a pancreatic NEN indicates that PSE is a safe and useful treatment in this indication.
